# Bedside selection of positive end-expiratory pressure by electrical impedance tomography in hypoxemic patients: a feasibility study

**DOI:** 10.1186/s13613-017-0299-9

**Published:** 2017-07-20

**Authors:** Nilde Eronia, Tommaso Mauri, Elisabetta Maffezzini, Stefano Gatti, Alfio Bronco, Laura Alban, Filippo Binda, Tommaso Sasso, Cristina Marenghi, Giacomo Grasselli, Giuseppe Foti, Antonio Pesenti, Giacomo Bellani

**Affiliations:** 1 Department of Emergency and Intensive Care, San Gerardo Hospital, Via Pergolesi 33, Monza, Italy; 2 Department of Pathophysiology and Transplantation, University of Milan, Via Festa del Perdono 7, Milan, Italy; 3 Department of Anesthesia, Critical Care and Emergency, Fondazione IRCCS Ca’ Granda Ospedale Maggiore Policlinico, Via Francesco Sforza 28, Milan, Italy; 4 Department of Medicine, School of Medicine and Surgery, University of Milan-Bicocca, Via Cadore 48, Monza, Italy

**Keywords:** EIT, PEEP, Overdistension, Recruitment

## Abstract

**Background:**

Positive end-expiratory pressure (PEEP) is a key element of mechanical ventilation. It should optimize recruitment, without causing excessive overdistension, but controversy exists on the best method to set it. The purpose of the study was to test the feasibility of setting PEEP with electrical impedance tomography in order to prevent lung de-recruitment following a recruitment maneuver. We enrolled 16 patients undergoing mechanical ventilation with PaO_2_/FiO_2_ <300 mmHg. In all patients, under constant tidal volume (6–8 ml/kg) PEEP was set based on the PEEP/FiO_2_ table proposed by the ARDS network (PEEP_ARDSnet_). We performed a recruitment maneuver and monitored the end-expiratory lung impedance (EELI) over 10 min. If the EELI signal decreased during this period, the recruitment maneuver was repeated and PEEP increased by 2 cmH_2_O. This procedure was repeated until the EELI maintained a stability over time (PEEP_EIT_).

**Results:**

The procedure was feasible in 87% patients. PEEP_EIT_ was higher than PEEP_ARDSnet_ (13 ± 3 vs. 9 ± 2 cmH_2_O, *p* < 0.001). PaO_2_/FiO_2_ improved during PEEP_EIT_ and driving pressure decreased. Recruited volume correlated with the decrease in driving pressure but not with oxygenation improvement. Finally, regional alveolar hyperdistention and collapse was reduced in dependent lung layers and increased in non-dependent lung layers.

**Conclusions:**

In hypoxemic patients, a PEEP selection strategy aimed at stabilizing alveolar recruitment guided by EIT at the bedside was feasible and safe. This strategy led, in comparison with the ARDSnet table, to higher PEEP, improved oxygenation and reduced driving pressure, allowing to estimate the relative weight of overdistension and recruitment.

**Electronic supplementary material:**

The online version of this article (doi:10.1186/s13613-017-0299-9) contains supplementary material, which is available to authorized users.

## Background

Acute respiratory distress syndrome (ARDS) is a relatively common and severe form of respiratory failure, characterized by massive non-cardiogenic pulmonary edema, with consequent loss of aeration in the alveolar spaces [1]. ARDS patients require intubation and mechanical ventilation as lifesaving procedures, and positive end-expiratory pressure (PEEP) is a key element of mechanical ventilation settings. Since both lower and higher PEEP levels may be associated with significant adverse consequences [2], personalized PEEP setting might be of cornerstone importance. Ideally, PEEP should optimize recruitment to improve oxygenation and reduce lung strain, without causing excessive overdistension, but controversy exists on the best bedside method to select PEEP. A common clinical approach is based on the severity of hypoxemia, relying on the use of PaO_2_/FiO_2_ tables [3]. Other approaches to select “personalized PEEP” are based on its effect on respiratory mechanics, focusing on plateau pressure [4], on stress index [5] or on transpulmonary pressure [6, 7]. These methods, however, do not provide consistent finding [8] and share the limitation of “lumping” into one measurement heterogeneous processes within the lung (i.e., recruitment, tidal opening–closing and overdistension [9, 10]) and of using surrogate rather than direct measures for lung recruitment induced by PEEP. In summary, bedside personalization of PEEP is still quite far from clinical practice. Randomized clinical trials have not shown clear benefit by indiscriminate application of high PEEP levels: Although a meta-analysis suggested that “higher” PEEP might be beneficial in moderate–severe ARDS [11], in everyday clinical practice clinicians still tend to apply relatively low PEEP levels even in severe ARDS patients [12].

In the present study, we hypothesized that the optimal PEEP level for each patient may be selected by assessing its efficacy in maintaining alveolar recruitment induced by a recruitment maneuver (RM). RMs are transient and voluntary increases in transpulmonary pressure that could reopen previously collapsed alveoli; they typically consist of application of continuous positive airway pressure of 30–50 cmH_2_O for 20–40 s, or transient increases in PEEP and/or inspiratory pressure, with a consequent increase in end-expiratory lung volume (EELV), decrease in lung strain and improvement in patient’s oxygenation [13, 14].

However, if RM is not followed by the application of adequate PEEP, EELV will progressively decrease over time (alveolar de-recruitment). At the opposite, RM plus adequate PEEP level will minimize de-recruitment and maintain sustained recruitment.

Electrical impedance tomography (EIT) is a noninvasive, radiation-free, bedside lung monitoring technique [15], that tracks real-time changes in regional lung ventilation and end-expiratory lung impedance (EELI), which closely correlate with EELV changes [16]. While several studies demonstrated the ability of EIT in assessing alveolar recruitment [17, 18], only a few used this method to guide therapy in humans [19]. The aim of the present study was to assess the feasibility of personalized PEEP selection based on its efficacy in stabilizing the EELV increase induced by a RM, using EIT as tool to monitor EELV changes. Moreover, we compared the effects of the selected PEEP on gas exchange, respiratory mechanics, hemodynamics and tidal recruitment/de-recruitment and overdistension with those induced by the application of PEEP levels selected according to PEEP/FiO_2_ tables. This comparator was chosen because of its large acceptance in the clinical practice and the frequent use of this approach as a control in other studies aimed at testing physiological-based methods to titrate PEEP [4, 6].

## Methods

The study was conducted in the general intensive care units of the university-affiliated hospitals San Gerardo, Monza and Fondazione IRCCS Ca’ Granda Ospedale Maggiore Policlinico, Milan, both in Italy. Institutional ethical committees of each institution approved the study, and informed consent was obtained according to local recommendations. Inclusion criteria were as follows: patient with acute hypoxemic respiratory failure (PaO_2_/FiO_2_ ratio ≤300 mmHg) of non-cardiogenic origin undergoing controlled mechanical ventilation with PEEP ≥ 5 cmH_2_O. Exclusion criteria were as follows: age <18 years, pregnancy, hemodynamic instability (requiring vasoactive drugs), presence of pneumothorax or lung emphysema, previous history of severe chronic obstructive pulmonary disease (GOLD III or IV), contraindications to the use of EIT (e.g., presence of pacemaker or automatic implantable cardioverter–defibrillator) and impossibility to place the EIT belt in the right position (e.g., presence of surgical wounds dressing). At enrollment, the following variables were collected: sex, age, predicted body weight (PBW), body mass index, Simplified Acute Physiology Score II (SAPS II) value at ICU admission, etiology of acute respiratory failure, diagnosis of ARDS and duration of intubation before enrollment. Vital status was recorded for all patients at ICU discharge.

### EIT monitoring

An EIT-dedicated belt equipped with 16 electrodes was placed around each patient’s chest at the fifth or sixth intercostal space and connected to a commercial EIT monitor (PulmoVista^®^ 500, Dräger Medical GmbH, Lübeck, Germany). EIT data were generated by application of small alternate electrical currents rotating around patient’s thorax, registered at 20 Hz and stored for offline analysis. When patients were ventilated with a ventilator able to communicate by serial protocol with the EIT device, airway pressure, flow and volume tracings were continuously recorded by EIT machine.

### Study protocol

Patients were deeply sedated (Richmond sedation scale −4 or −5) and paralyzed and mechanical ventilation was set in volume controlled according to the ARDSnet guidelines, as follows: Vt = 6–8 mL/kg of PBW; plateau pressure lower than 30 cmH_2_O; respiratory rate was targeted to a pH value of 7.30–7.45; PEEP and FiO_2_ were set according to the lower PEEP/higher FiO_2_ table [3], targeting a partial arterial oxygen tension = 55–80 mmHg or SpO_2_ = 88–95%.

**Fig. 1:**
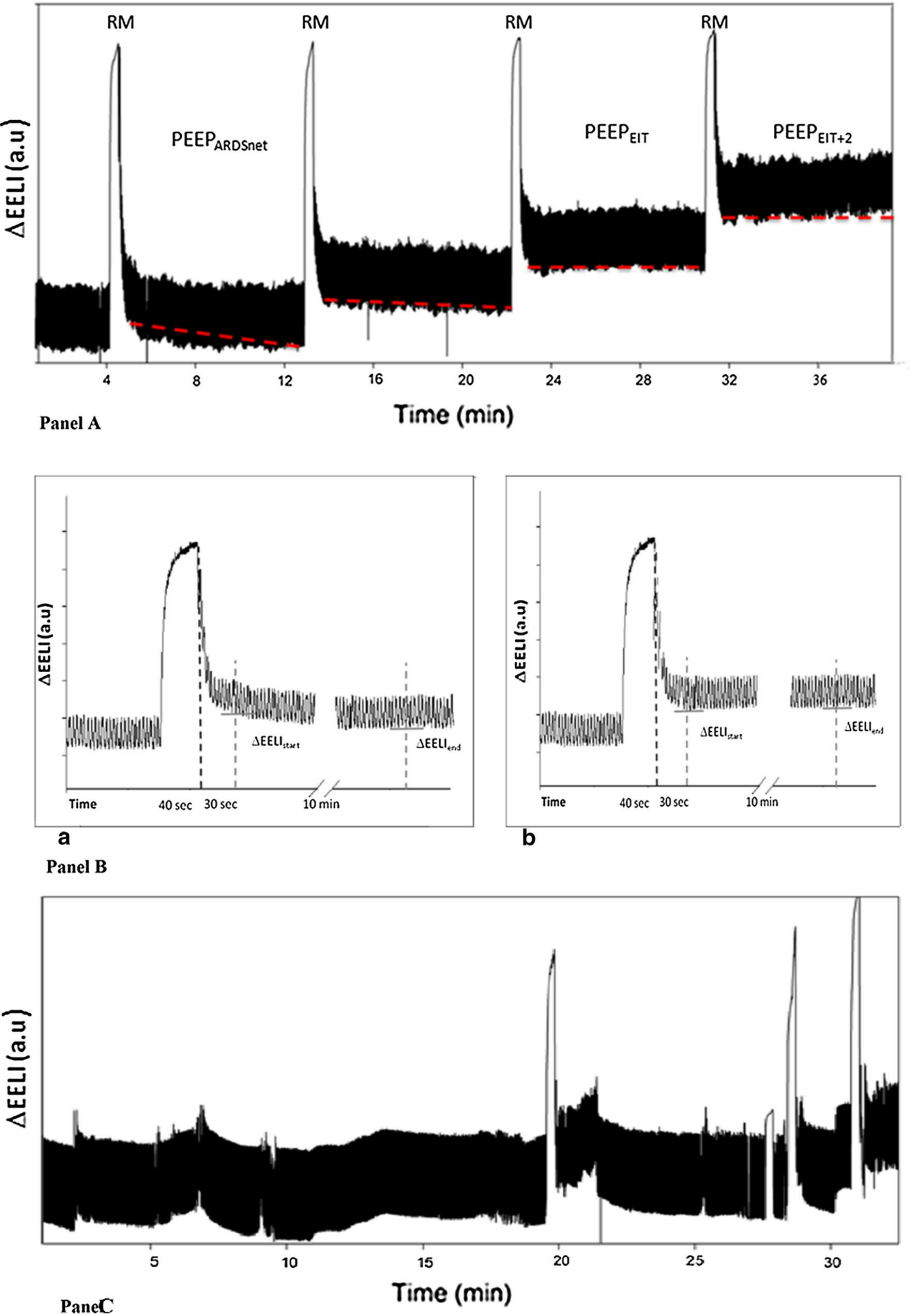
PEEP selection by EIT (*Panel A* and *B*): After a baseline phase lasting 20 min (PEEP_ARDSnet_), a RM was performed (whose duration is shortened in the image for clarity purposes); end-expiratory lung impedance variation (∆EELI) was measured after 30 s (∆EELIstart) and after 10 min (∆EELIend); if ∆EELIend decreased more than 10% of ∆EELIstart, a new RM was performed, and PEEP increased by 2 cmH_2_O. This was repeated until ∆EELIend decreased less than 10% of ∆EELIstart, or up to maximum PEEP level of 18 cmH_2_O (PEEP_EIT_). A new RM was performed and PEEP increased by 2 cmH_2_O from PEEP_EIT_ (PEEP_EIT+2_). Unstable EELI track (*Panel C*): an example of unstable EELI track

Then, study protocol, shown in Fig. 1A, consisted of three consecutive steps:

1. Baseline phase (20 min): volume-controlled ventilation set as previously described (PEEP_ARDSnet_)
2. PEEP_EIT_ selection phase, which included:
  2.1. Application of a RM, with a positive pressure of 40 cmH_2_O for 40 s.
  2.2. Measure of EELI variation (∆EELI) 30 s (∆EELIstart) and 10 min (∆EELIend) after the RM.
  2.3. Calculation of ∆EELI change: If ∆EELIend decreased more than 10% of ∆EELIstart (Fig. 1B-a), a new RM was performed, and PEEP increased by 2 cmH_2_O
  2.4. If ∆EELI decreased less than 10% of ∆EELIstart (Fig. 1B-b), PEEP was left unchanged.
    If, after the first RM, ∆EELI increased less than 10% of ∆EELIstart, PEEP was reduced by 2 cmH_2_O, every 10 min, until a decrease in ∆EELI of 10% or more was observed.
  2.5. The first three points of STEP 2 were repeated until ∆EELI change fulfilled point 2.4 requirement, up to a maximum PEEP level of 18 cmH_2_O. (PEEP_EIT_).
3. PEEP_EIT+2_ phase, lasting 10 min: A new RM was performed and PEEP increased by 2 cmH_2_O from PEEP_EIT_ (Fig. 1A).

The reliability of bedside-derived calculation of relative changes in ∆EELIstart and ∆EELIend was also verified offline (Additional file 1: Figure E2).

At the end of each phase (i.e., PEEP_ARDSnet_, PEEP_EIT_, PEEP_EIT+2_), arterial blood gases were collected, mean arterial pressure, central venous pressure and heart rate were recorded and end-inspiratory and end-expiratory occlusions performed, lasting about 3 s. Then, from offline analysis of ventilation tracings, plateau pressure and total PEEP (including intrinsic PEEP) were measured. Driving pressure was calculated as plateau pressure − total PEEP and respiratory system compliance as Vt/driving pressure.

Tidal volume was held constant during the protocol. PEEP was increased up to a maximum level of 18 cmH_2_O; the protocol foresaw a decrease in Vt if a plateau pressure higher than 30 was reached. This was, however, never the case in our patients. Clinically set FiO_2_ was left unchanged throughout the study. However, from the value of PaO_2_ measured at PEEP_EIT_, we calculated the “predicted FiO_2_” value at which the PaO_2_ in PEEP_EIT_ step would have been equal to ARDSnet step: “predicted FiO_2_” = [PaO_2_/FiO_2_ARDSnet/PaO_2_/FiO_2_EIT × FiO_2_ ARDSnet]. Since PaO_2_ is not linearly related to FiO_2_, this calculation, not previously validated, should be interpreted with caution [20].

We also calculated the PEEP level that would have resulted from the Express protocol [4] as 30 cmH_2_O minus driving pressure (at PEEP_EIT_).

### EIT data

Besides the previously described bedside evaluation of EIT tracings used to titrate PEEP, EIT data were also analyzed offline to derive further parameters. The whole PEEP titration protocol was acquired as sequential EIT files lasting 5 min with the same baseline reference. EIT data analyses were performed after identification of a sequence of breaths deemed as representative (i.e., stable Vt and EELI) at the end of each phase. We defined four horizontal same-size contiguous layers [ventral (V), middle-ventral (MV), middle-dorsal (MD), dorsal (D)], encompassing the entire field of view (Additional file 1: Figure E1), and from offline analyses (performed by EIT Data Analysis Tool 6.0, Dräger Medical GmbH, Lübeck, Germany) of average raw EIT data of the selected breaths, the following variables were obtained for each study phase:

- Regional compliance: We obtained regional distribution of Vt during inspiratory occlusion (Vt_%_) expressed as percentage of its global value, and then we calculated regional compliance as Vt_%_/100 * compliance.
- Alveolar hyperdistension and collapse: As Vt correlates well with local impedance lung changes [21–24], pixel-by-pixel compliance was calculated as ∆impedance/(plateau pressure—PEEP). Alveolar overdistension and collapse was then computed as previously described by Costa et al. [25]. Finally, alveolar hyperdistension and collapse was calculated as the sum of alveolar hyperdistension and collapse expressed as percentage value.
- The amount of recruited volume was calculated as the difference between actual EELV change (measured by EIT) minus the product of compliance at lower PEEP and the PEEP change, as previously described: Recruited volume = ∆EELV − [compliance_ARDSnet_ * (PEEP_EIT_ − PEEP_ARDSnet_)] [16].

### Statistical analysis

In the study population, we expected a PaO_2_/FiO_2_ ratio = 199 ± 57 [16]. In order to detect an increase in PaO2/FiO2 ratio of 25%, in a crossover design, we estimated that 12 patients would be necessary. Since the feasibility of the technique was unknown, we increased this by 30%, obtaining a final sample size of 16 patients. Differences between variables obtained during each study phase were tested by one-way analysis of variance (ANOVA) for repeated measures, or by one-way repeated measures ANOVA on ranks for non-normally distributed variables; post hoc comparisons were made by the Bonferroni’s method. Comparisons between two groups of normally distributed variables were made by independent samples *t* test, while non-normally distributed variables were compared by Mann–Whitney *U* test. A level of *p* < 0.05 (two-tailed) was considered as statistically significant. Normally distributed data are indicated as mean ± standard deviation, while median and interquartile range [IQR] are used to report non-normally distributed variables. Statistical analyses were performed by SigmaPlot 11.0 (Systat Software Inc., San Jose, CA).

## Results

### Patients’ characteristics

Patients’ main characteristics are summarized in Table 1. Patients were 66 ± 11 years old and 14 (87%) were men. On the day of the study, 12 patients (75%) fulfilled ARDS criteria. Fourteen patients (87%) were enrolled within a week from intubation. The diagnosis at ICU admission was pneumonia in ten patients (62%), thoracic trauma in three patients (19%) and septic shock in three patients (19%). Three patients (19%) died during their hospital stay.

**Table 1:** Patients’ main characteristics

Patient #	Age (years)	Sex	Body mass index (Kg/m^2^)	SAPS II score	PaO_2_/FiO_2_ (mmHg)	Diagnosis at admission	ARDS	Days of intubation before enrollment	ICU outcome
1	59	M	26	39	121	Thoracic trauma	Y	1	Survive
2	67	M	29	33	114	Pneumonia	Y	3	Survive
3	67	M	24	34	236	Pneumonia	Y	4	Survive
4	55	M	26	46	170	Pneumonia	Y	15	Survive
5	75	F	31	47	84	Pneumonia	Y	2	Survive
6	80	F	28	78	145	Pneumonia	Y	1	Dead
7	63	M	29	45	140	Pneumonia	N	2	Survive
8	41	M	34	33	209	Pneumonia	Y	3	Survive
9	79	M	24	44	97	Pneumonia	Y	1	Survive
10	69	M	25	35	279	Thoracic trauma	Y	3	Survive
11	64	M	26	48	238	Thoracic trauma	N	3	Dead
12	63	M	28	42	104	Pneumonia	Y	3	Dead
13	56	M	37	39	86	Pneumonia	Y	2	Survive
14	88	M	26	38	210	Septic shock	Y	1	Survive
15	68	M	26	51	196	Septic shock	N	19	Survive
16	59	M	29	31	132	Septic shock	N	6	Survive
Mean ± SD	66 ± 11	2 F	28 ± 4	43 ± 11	160 ± 60	10 pneumonia, 3 thoracic trauma, 3 septic shock	12 Y	4 ± 5	3 dead

SAPS, Simplified Acute Physiologic Score; ARDS, acute respiratory distress syndrome; ICU, intensive care unit

### Feasibility of setting PEEP by EIT-based evidence of sustained recruitment

We enrolled 16 patients: Clinical PEEP level at study enrollment was 11 ± 3 cmH_2_O; EELI tracing could successfully detect the PEEP level associated with sustained recruitment in 14 (87%) patients; of these 14 patients, 11 (78%) fulfilled ARDS; the distribution of tidal volume during PEEP_ARDSnet_ phase was 52 ± 11% in right lung and 48 ± 10% in left lung (*p* = 0.557).

In two patients (13%), EIT tracings could not be used due to the lack of stability of the EELI signal (Fig. 1C), and thus, their data were excluded from further analysis.

PEEP_EIT_ was significantly higher than PEEP_ARDSnet_ (13 ± 3 vs. 9 ± 2 cmH_2_O, *p* < 0.001), and the correlation between them was significant, but loose (*R* ^2^ = 0.36, *p* = 0.022). The mean number of stepwise changes in PEEP performed between PEEP_ARDSnet_ and PEEP_EIT_ phases was 2 ± 1, and the total duration of time required to arrive at the PEEP_EIT_ was 48 ± 12 min. The largest PEEP variations performed were 6 cmH2O in two patients and 10 cmH_2_O in one patient.

### Effects of PEEP selection on oxygenation, respiratory mechanics and hemodynamics

**Fig. 2:**
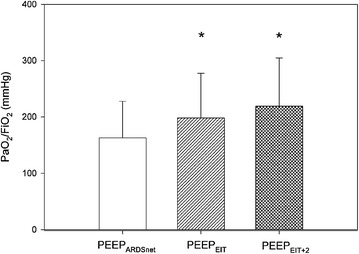
PaO_2_/FiO_2_ ratio in all study phases. It significantly improved in both PEEP_EIT_ and PEEP_EIT+2_ phases compared with PEEP_ARDSnet_. *p < 0.05 compared with PEEP_ARDSnet_ phase

**Fig. 3:**
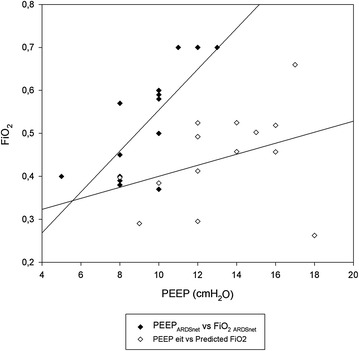
Correlation between PEEP and FiO_2_ set according to ARDSnet and EIT: As expected, there was a strong correlation between PEEP_ARDSnet_ and FiO_2_ set according to ARDSnet table (*R* ^2^ = 0.80, *p* < 0.001); on the contrary, no significant association was observed between PEEP_EIT_ and predicted FiO_2_ (*R* ^2^ = 0.12, *p* = 0.217)

Ventilation tracings were continuously recorded by EIT in 9/14 patients; FiO_2_ during all study phases was kept stable at 0.5 ± 0.1; PaO_2_/FiO_2_ ratio improved during both PEEP_EIT_ and PEEP_EIT+2_ phases compared with PEEP_ARDSnet_ (Fig. 2), but no significant changes occurred between PEEP_EIT_ and PEEP_EIT+2_ phases (*p* = 0.121). The predicted FiO_2_ at PEEP_EIT_ would have been significantly lower compared with the ARDSnet table (0.44 ± 0.1 vs. 0.53 ± 0.1, *p* ≤ 0.001). Moreover, while (as expected) there was a strong correlation between PEEP_ARDSnet_ and FiO_2_ ARDSnet, no significant association was observed between PEEP_EIT_ and predicted FiO_2_ (Fig. 3). At PEEP_EIT_ levels, compliance did not significantly change (*p* = 0.097), whereas the driving pressure was significantly reduced in PEEP_EIT_ phase compared with ARDSnet phase (Table 2), albeit with a probably modest clinical relevance (range between −2 and 0 cmH_2_O). The PEEP level theoretically achieved with Express trial approach was significantly higher than PEEP_EIT_ (20.6 ± 1.9 vs. 13.1 2.9 cmH_2_O, *p* < 0.001), without any significant association (*R*_2_ = 0.002).

**Table 2:** Global and regional respiratory system compliance in all study phases

	PEEP_ARDSnet_	PEEP_EIT_	PEEP_EIT+2_	*p* value
Driving pressure (cmH_2_O)	10.2 ± 1.9	9.3 ± 1.9*	9.7 ± 2.5	0.035
Compliance (ml/cmH_2_O)	44.6 ± 11	49.5 ± 12	49.5 ± 17	0.097
Compliance_V_ (ml/cmH_2_O)	6.9 ± 3	5.0 ± 2*	4.1 ± 2*	<0.01
Compliance_MV_ (ml/cmH_2_O)	24.3 ± 9	24.9 ± 10	23.8 ± 12	0.873
Compliance_MD_ (ml/cmH_2_O)	6.9 ± 6	14.6 ± 6*	16.1 ± 7*	<0.001
Compliance_D_ (ml/cmH_2_O)	3.2 ± 2	4.8 ± 5	5.1 ± 5*	<0.05

V, ventral; MV, middle-ventral; MD, middle-dorsal; D, dorsal

* *p* < 0.05 compared with PEEP_ARDSnet_ phase

No significant hemodynamic changes were observed during all study phases (Table 3); PaCO_2_ remained stable over all study phases (45 ± 7, vs. 46 ± 9, vs. 47 ± 9 mmHg, *p* = 0.1).

**Table 3:** Hemodynamics during all study phases

	PEEP_ARDSnet_	PEEP_EIT_	PEEP_EIT+2_	*p* value
Mean arterial pressure (mmHg)	77 ± 10	73 ± 7	75 ± 11	0.079
Heart rate (bpm)	86 ± 16	83 ± 18	87 ± 18	0.066
Central venous pressure (mmHg)	12 ± 5	13 ± 6	13 ± 6	0.214

### Homogeneity and regional mechanics

**Fig. 4:**
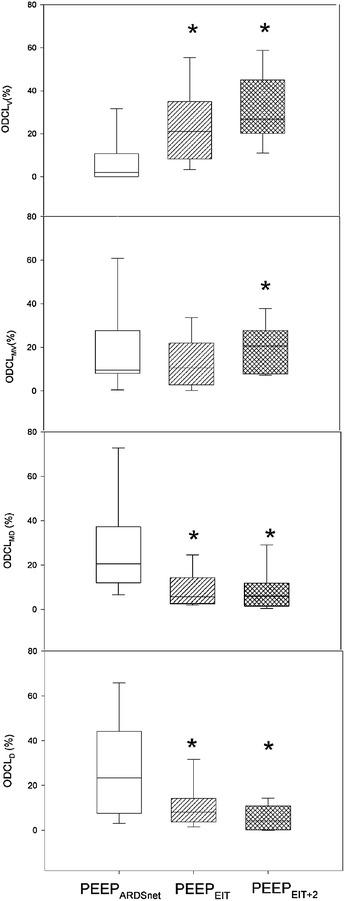
Regional alveolar hyperdistension and collapse distribution in all study phases. Alveolar hyperdistension and collapse was significantly reduced in dependent lung layers and significantly increased in non-dependent lung layers compared with PEEP_ARDSnet_ in both PEEP_EIT_ and PEEP_EIT+2_ phases. Furthermore, in middle-ventral lung layers alveolar hyperdistension and collapse was significantly higher in PEEP_EIT+2_ phase compared with PEEP_ARDSnet_, but did not change in PEEP_EIT_ step. *p < 0.05 compared with PEEP_ARDSnet_ phase

**Fig. 5:**
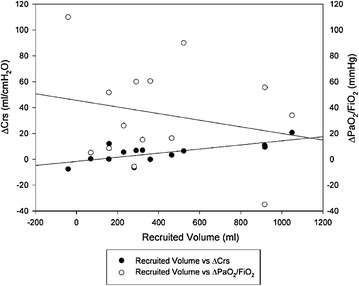
Correlations between recruited volume, compliance and oxygenation. The amount of recruited volume did not correlate with oxygenation improvement (*R* ^2^ = 0.04, *p* = 0.448), whereas it correlated with the improvement in respiratory system compliance (*R* ^2^ = 0.50, *p* < 0.01)

Regional compliance was reduced in ventral lung layer, and it improved in middle-dorsal lung layers, during both PEEP_EIT_ and PEEP_EIT+2_ phases compared with PEEP_ARDSnet_. In dorsal layer, compliance improved in PEEP_EIT+2_ phase, while no significant changes in middle-ventral layer were observed (Table 2). Interestingly, regional alveolar hyperdistension and collapse was significantly reduced in dependent lung layers and significantly increased in non-dependent lung layers during both PEEP_EIT_ and PEEP_EIT+2_ compared with PEEP_ARDSnet_. Furthermore, in middle-ventral lung layers alveolar hyperdistension and collapse was significantly higher in PEEP_EIT+2_ phase than in PEEP_ARDSnet_, but did not change in PEEP_EIT_ step (Fig. 4). The recruited volume at PEEP_EIT_ was 306 (159–522) ml. The amount of recruited volume did not correlate with oxygenation improvement (Fig. 5), whereas it correlated with changes in respiratory system compliance (*R* ^2^ = 0.50, *p* < 0.01, Fig. 5) and with the decrease in driving pressure (*R* ^2^ = 0.36, *p* < 0.05). No significant correlation was observed between oxygenation improvement and driving pressure reduction (*R* = 0.26, *p* = 0.36).

## Discussion

The main findings of this study can be summarized as follows: Bedside PEEP setting based on sustained recruitment following a RM as visualized by EIT was feasible in the majority of patients with acute hypoxemic respiratory failure (most of whom fulfilled ARDS criteria). This method invariably led to the application of higher PEEP levels in comparison with the commonly used ARDSnet table and was associated with improved oxygenation. Furthermore, EIT allowed to disclose and quantitate the presence of regional overdistension associated with PEEP increase. PEEP setting in hypoxemic respiratory failure and ARDS remains controversial. A meta-analysis on three large randomized trials [3] showed that higher PEEP could be beneficial in more severe ARDS patients, but no indication was provided on how to titrate this higher PEEP: Indeed, while in two studies an oxygenation-based criterion was used, in the third trial a respiratory mechanics-based method was used. Later, a secondary analysis by Goligher et al. [26] showed that the benefit of higher PEEP on mortality was limited to the patients who had an oxygenation improvement, likely indicating the presence of recruitment. Even if the aforementioned paper does not necessarily proof a cause relationship effect (i.e., that higher PEEP prevented death in recruiters), we reasoned that it would have been desirable to have a method to set PEEP directly targeting alveolar recruitment combined with prevention of de-recruitment, and we focused on EIT as a bedside noninvasive tool. While the ability of EIT in assessing lung recruitment has been previously shown, most studies were conducted on animal models and/or explored the effects of relatively high PEEP changes (in the order of 10 cmH_2_O) [27, 28]. On the contrary, we aimed to test the feasibility of a specific protocol, with a “fine tuning” of PEEP in steps of 2 cmH_2_O. Since we expected that in most patients the EIT-based approach would have led to increase in PEEP from the NIH table, we added PEEP_EIT+2_ phase, in order to establish the effects on oxygenation and respiratory mechanics of a further increase in PEEP above the level set by EIT. The method proposed appeared feasible in most patients: This is encouraging in prospect of future evaluation of the protocol in a clinical setting. A PEEP increase will always be associated with an increment of EELV, even if no recruitment occurs, simply because of the expansion of ventilated alveoli. Hence, to dissect these two phenomena, we took advantage of RM. Although available literature shows that RM does not impact outcome, it also shows that it is safe and devoid of major complications [29]. Grasso et al. [30] showed how RMs are unlikely to benefit patients with more than 5 days of ARDS; plus, Borges et al. [31, 32] obtained best results in terms of recruitability with RMs involving stepwise increases in PEEP compared with sustained inflation methods; however, we included in the algorithm RMs more as “diagnostic” tools to exploit potential for lung recruitment, rather than therapeutic measures; for this reason, we did not focus on a specific category of patients and we chose the most simple and immediate method of recruitment, usually used in our clinical practice.

As a reference method, we used the ARDSnet table, one of the most frequently applied methods. Not surprisingly, EIT led to an increase in PEEP in all subjects: This finding is not surprising, since the ARDSnet table has been used to set PEEP in the control group of all studies testing “higher” PEEP strategies.

In the majority of patients, we found that the protocol was feasible: It led to the univocal identification of a PEEP level associated with sustained recruitment after a RM, without exceeding the upper safety limit set to 18 cmH_2_O. In the majority of patients, the PEEP changes were within a relatively narrow range (mean 4 cmH_2_O) and hence safe to apply. Despite relatively small, however, these changes were clinically relevant, leading to improvement in oxygenation. It was likely due to lung recruitment and increased lung size (as indicated also by the positive correlation between these two variables), and this might possibly lead to a decreased injury from mechanical ventilation. Interestingly, these improvements were not due to the increase in PEEP per se, since a further increase in PEEP above PEEP_EIT_ was not associated with further improvement in gas exchange and respiratory mechanics.

The global change in compliance between PEEP_ARDSnet_ and PEEP_EIT_ phases was the net result of two opposed changes in regional compliance: increasing in the dorsal layers (likely due to recruitment) and decreasing in the ventral layers (likely due to overdistension). We cannot exclude that part of the improvement in the compliance of dorsal layers might be due to the presence of intratidal recruitment; however, this effect was unlikely since previus studies showed that increasing PEEP leads to a decrease in intratidal recruitment [2]. FiO_2_ was kept stable in all study phases (0.5 ± 0.1); in this way, we avoided erroneous estimation of alveolar collapse and recruitment due to low alveolar oxygen concentration [31, 33]. We believe that this result further underlines the need for a regional real-time monitoring of the distribution of ventilation, which could prompt a decrease in tidal volume until overdistension of ventral regions drops back to baseline.

This study has some limitations that need to be acknowledged. EIT measurement encompasses only a cross-sectional slice of 5–10 cm of the thorax, and we assumed that other lung regions behave similarly. However, previous studies on similar patient populations showed that ∆EELV measured by EIT well represents the entire lung [34]. The study population was relatively small, but large enough to test the feasibility, safety and efficacy on selected physiological endpoints. Our results do not provide any evidence that a strategy aimed at obtaining stable recruitment leads to a decreased lung injury, but show that such aim can be achieved and that the overdistension also induced by PEEP can be simultaneously monitored and could be used to further adjust ventilation (e.g., reducing tidal volume). Therefore, these results pave the way to a larger study, aimed at assessing whether this approach to PEEP setting leads to benefit in outcome.

Finally, we have not limited our population to ARDS patients: This choice was on one the hand pragmatic, facilitating the enrollment of patients, but on the other hand it also acknowledges the fact that alveolar de-recruitment is not unique of ARDS and that mechanical ventilation can be a challenge also in acutely hypoxemic patients. As this study aimed to test the feasibility of the method, we exclude that the inclusion of non-ARDS patient introduced a significant bias, while we are uncertain of what would be the best approach in an outcome study.

## Conclusions

This study shows that, in a cohort of patients with acute hypoxemic respiratory failure undergoing lung protective ventilation, a PEEP selection strategy aimed at maximizing alveolar recruitment and preventing de-recruitment, guided by EIT at the bedside, is feasible, simple and safe, leading to systematically higher PEEP values than the ARDSnet table, with positive effects on gas exchange and respiratory system mechanical properties. This strategy also allows estimating the relative weight of overdistension and recruitment following a PEEP change. These results do not necessarily imply that benefits of recruitment achieved out weight the negative effects induced by overdistension and larger study are required to elucidate if this strategy could also lead to improved clinical outcome.

## Additional file

**Additional file 1: Figure E1.** Definition of the layers. We defined four horizontal same-size contiguous layers [ventral (V), middle-ventral (MV), middle-dorsal (MD), dorsal (D)], encompassing the entire field of view. **Figure E2.** Average drop of EELI, obtained from offline tracing analysis, occurring between 30 s after the recruitment manoeuver (DEELI start) to ten minutes after, for the ARDSnet step, for the first step of PEEP titration (Step #1) and for PEEP_EIT_. Dashed line indicates the 10% drop which we used as a cutoff to set PEEP based on EIT

## Abbreviations

ARDS: acute respiratory distress syndrome
EELI: end-expiratory lung impedance
EELV: end-expiratory lung volume
EIT: electrical impedance tomography
ICU: intensive care unit
PBW: predicted body weight
PEEP: positive end-expiratory pressure
RM: recruitment maneuver
SAPS II: Simplified Acute Physiology Score II
