# Supplementary material for: Bedside selection of positive end-expiratory pressure by electrical impedance tomography in hypoxemic patients: a feasibility study
Source: Ann Intensive Care. 2017 Jul 20;7:76. doi: 10.1186/s13613-017-0299-9 (PMC5519511; doi:10.1186/s13613-017-0299-9)
Supplement: Supplementary file 1 — Additional file 1: Figure E1. Definition of the layers. We defined four horizontal same-size contiguous layers [ventral (V), middle-ventral (MV), middle-dorsal (MD), dorsal (D)], encompassing the entire field of view. Figure E2. Average drop of EELI, obtained from offline tracing analysis, occurring between 30 s after the recruitment manoeuver (DEELI start) to ten minutes after, for the ARDSnet step, for the first step of PEEP titration (Step #1) and for PEEPEIT. Dashed line indicates the 10% drop which we used as a cutoff to set PEEP based on EIT [file 13613_2017_299_MOESM1_ESM.docx]

**Bedside selection of positive end-expiratory pressure by Electrical Impedance Tomography in hypoxemic patients: a feasibility study.**

SUPPLEMENTAL DATA


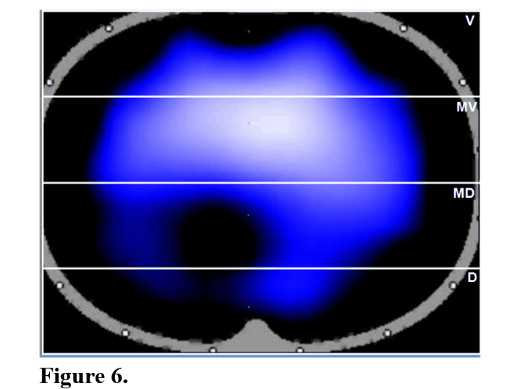


**Figure E1: Definition of the layers.**  We defined four horizontal same-size contiguous layers [ventral (V), middle-ventral (MV), middle-dorsal (MD), dorsal (D)], encompassing the entire field of view.

**Figure E2**: Average drop of EELI, obtained from offline tracing analysis, occurring between 30’ after the Recruitment Manouver (DEELI start) to ten minutes after, for the ARDSnet step, for the first step of PEEP titration (Step #1) and for PEEP_EIT_ . Dashed line indicates the 10% drop which we used as a cut-off to set PEEP based on EIT.
